# Supplementary material for: Causal interplay between lactose intolerance and gut microbiota: a combined bidirectional Mendelian randomization and in vivo validation study
Source: Front Nutr. 2026 Jun 1;13:1803337. doi: 10.3389/fnut.2026.1803337 (PMC13265576; doi:10.3389/fnut.2026.1803337)
Supplement: Supplementary file 1 [file Data_Sheet_1.zip › supplementary materials/Forward/forest plot/ebi-a-GCST90027704.finngen_R12_E4_LACTONAS.pdf]

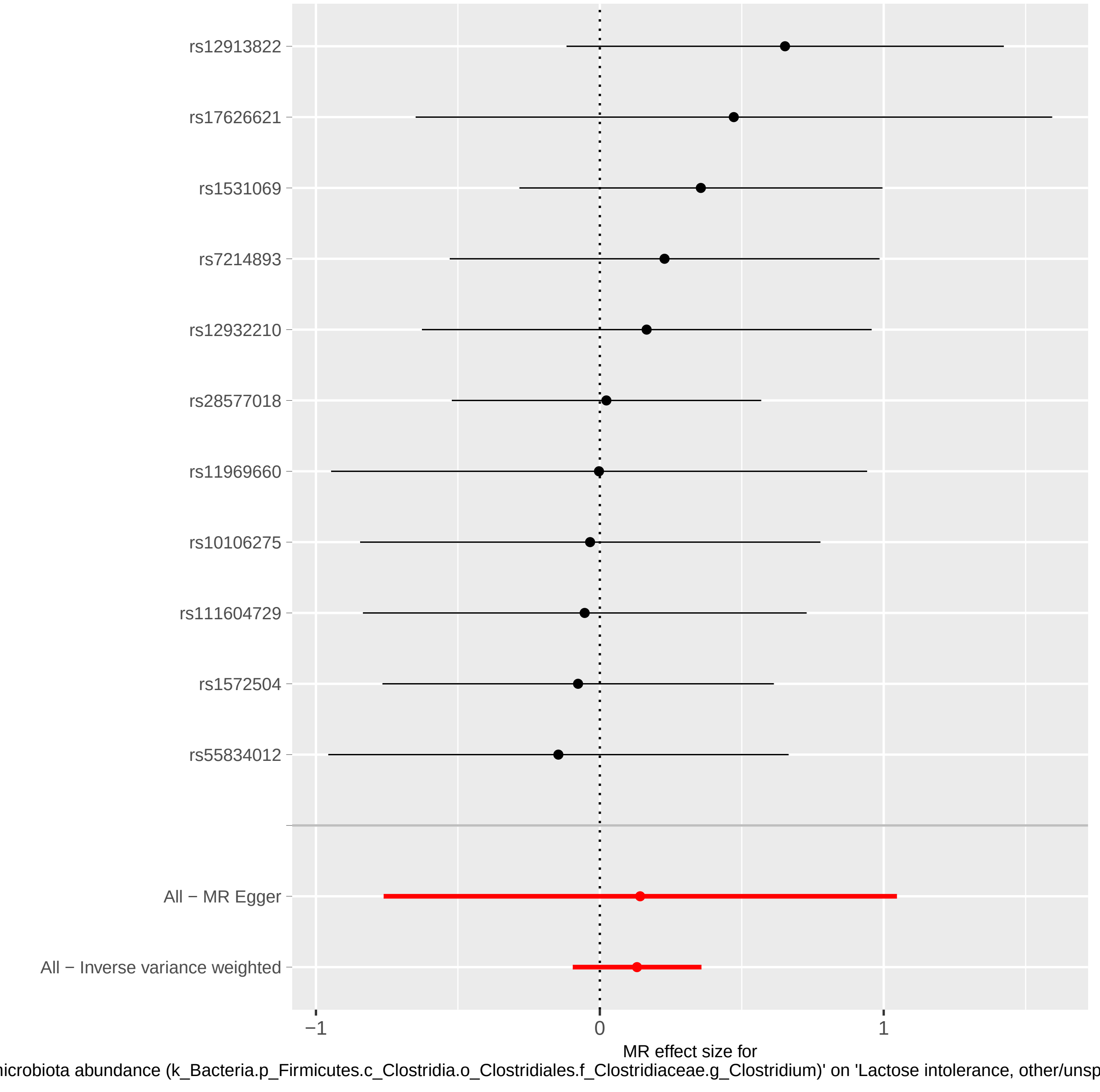
rs12913822
rs17626621
rs1531069
rs7214893
rs12932210
rs28577018
rs11969660
rs10106275
rs111604729
rs1572504
rs55834012
All − MR Egger
All − Inverse variance weighted
−1
0
1
MR effect size for
nicrobiota abundance (k_Bacteria.p_Firmicutes.c_Clostridia.o_Clostridiales.f_Clostridiaceae.g_Clostridium)' on 'Lactose intolerance, other/unsp
